# Supplementary material for: Can rapid approaches to qualitative analysis deliver timely, valid findings to clinical leaders? A mixed methods study comparing rapid and thematic analysis
Source: BMJ Open. 2018 Oct 8;8(10):e019993. doi: 10.1136/bmjopen-2017-019993 (PMC6194404; doi:10.1136/bmjopen-2017-019993)
Supplement: Supplementary file 1 [file bmjopen-2017-019993supp001.pdf]

Summary Template

Home Birth Service Exploratory Review Summary Template
**PREPARED BY**:
**DATE**:
**DATA TYPE** (document, interview transcript, focus group transcript):
**FOR INTERVIEWS AND FOCUS GROUPS:**<br>***PARTICIPANT IDENTIFIER/S***<br>***PARTICIPANT ROLE***<br>***PARTICIPANT ROLE IN THE HBS***
**FOR DOCUMENTS:**<br>**TITLE**<br>**DATE OF PRODUCTION**<br>**AUTHOR**
**RATIONALE FOR IMPLEMENTING HBS (POLICY, EVIDENCE, OTHER)**<br>**What was the problem, what was going wrong?**<br>**What was the vision** (envisaged outputs, outcomes, impact)
**TRAINING:**
**PROGRAMME THEORY/LOGIC MODEL:**<br>***INPUTS*** *(resources, people)*<br>***ACTIVITIES*** *(what workers do, e.g. promotional work, clinical care)*<br>***OUTPUTS*** *(what activities deliver, e.g. women informed about the HBS, women receive intrapartum care at home)* ***and OUTCOMES*** *(results of the outputs, e.g. women book with the HBS, women give birth at home)*<br>***IMPACT/GOAL*** *(overall aims of programme, e.g. home birth booking rate increases, home birth rate increases)*
**FACILITATORS of IMPLEMENTATION**<br>Planning/process<br>People<br>Culture<br>Money<br>Organisation/bureaucracy<br>Evidence/policy<br>Other
**BARRIERS TO IMPLEMENTATION AND SOLUTIONS**<br>Planning/process<br>People<br>Culture<br>Money<br>Organisation/bureaucracy<br>Evidence/policy<br>Other
**ROUTINELY GATHERED DATA** (HOW,WHERE, WHEN, WHO COLLECTED, WHERE DATA HELD)
**KEY DOCUMENTS WE SHOULD INCLUDE** (e.g. service specification)
**OTHER IMPORTANT OBSERVATIONS**
**IMPORTANT QUOTATIONS**
**REFLECTIONS ON THE DATA COLLECTION EPISODE**
